# External Control of the *GAL Network* in *S. cerevisiae*: A View from Control Theory

**DOI:** 10.1371/journal.pone.0019353

**Published:** 2011-04-29

**Authors:** Ruoting Yang, Scott C. Lenaghan, John P. Wikswo, Mingjun Zhang

**Affiliations:** 1 Department of Mechanical, Aerospace and Biomedical Engineering, The University of Tennessee, Knoxville, Tennessee, United States of America; 2 Vanderbilt Institute for Integrative Biosystems Research and Education, Departments of Biomedical Engineering, Molecular Physiology & Biophysics, and Physics & Astronomy, Vanderbilt University, Nashville, Tennessee, United States of America; Fondazione Telethon, Italy

## Abstract

While there is a vast literature on the control systems that cells utilize to regulate their own state, there is little published work on the formal application of control theory to the external regulation of cellular functions. This paper chooses the *GAL* network in *S. cerevisiae* as a well understood benchmark example to demonstrate how control theory can be employed to regulate intracellular mRNA levels via extracellular galactose. Based on a mathematical model reduced from the *GAL* network, we have demonstrated that a galactose dose necessary to drive and maintain the desired *GAL* genes' mRNA levels can be calculated in an analytic form. And thus, a proportional feedback control can be designed to precisely regulate the level of mRNA. The benefits of the proposed feedback control are extensively investigated in terms of stability and parameter sensitivity. This paper demonstrates that feedback control can both significantly accelerate the process to precisely regulate mRNA levels and enhance the robustness of the overall cellular control system.

## Introduction

The complexity of nested feedback control loops associated with complex biological systems creates many challenges to understanding biological systems. At the same time, the need for precise control of gene expression is increasing in fields such as genetics, cell and molecular engineering, and in disease treatment via gene expression control. A straightforward application for control of gene expression might be to generate a maximum output in bioreactor-based systems. The outputs from this type of application would typically by specific proteins of interest for biopharmaceuticals. The approach can be further applied to create a system in which the control of a gene network may be coupled with the expression of a recombinant protein.

On the other hand, systems biologists have built numerous mathematical models for gene and protein networks that cells utilize to control themselves [1]. The applications of such models have gone beyond simple qualitative understanding of network dynamics. Some models have been used in synthetic biology to affect metabolism and eventually control the biological processes toward desired outputs [2]. The full advantages of control theory, however, have not been realized, and there is little work on external control of cellular functions such as gene expression.

From a control theory perspective, control techniques used for complex engineering systems should in principle be applicable to the regulation of cellular systems. There are in general two potential challenges. First, it is not easy to find simple mathematical models for cellular system control. Most existing cellular mathematical models are not formulated in a standard control system framework. Typically, they lack global feedback loops from outputs to dynamically adjust the control dosage. Second, model parameters are usually very sensitive due to large uncertainties. The resulting control algorithm often becomes fragile, and the control system can remain stable only under small disturbances. Open-loop constant controls – for example, the gene knockdown or overexpression technique for yeast performed without adjusting dosages in real-time in response to the observed changes – are vulnerable to disturbances and individual heterogeneity.

It is desirable to have model-based, fine-tuned external control approaches to precisely regulate a reverse-engineered target. Unfortunately, achieving a reliable feedback control remains a challenge for cellular systems. Recently, Cantone *et al.* developed the first reverse-engineering benchmark system [3], which is based on the GAL network using real-time image feedback. Control engineers have begun to apply closed-loop control ideas based on this benchmark [4], [5].

In this paper we have employed model reduction techniques to simplify the complex GAL network, and further demonstrate that the simplified model is favorable for control system design. We also present the benefit of closed-loop system design. Our goal is to demonstrate the usefulness of control theory for precise external regulation of cellular systems and to open discussions in the field about possible future benefits of more advanced theoretical control.

**Figure 1:**
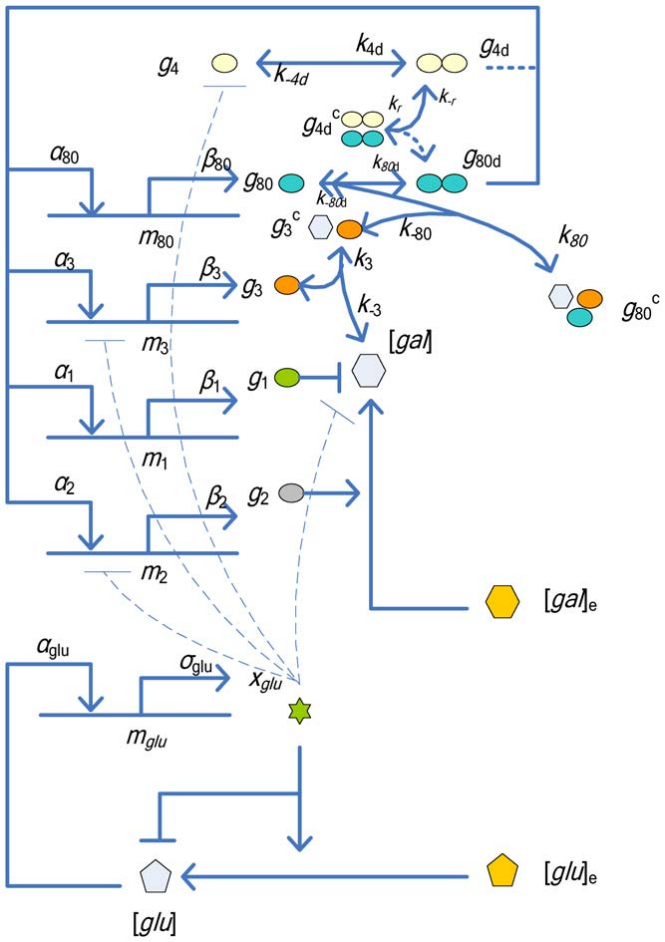
The gene regulatory network for galactose utilization (redrawn from Bennett *et al.* (2008) [**12**]). The extracellular inputs are [*gal*]_e_ and [*glu*]_e_. The natural output of the network is *a group of proteins*. The *GAL*4 protein *g*_4_ binds to upstream activation sites and activates the regulatory genes in the galactose network. The *GAL*80 gene inhibits the inducing effects of *GAL*4 and thereby provides negative feedback in the system. *GAL*3 enhances expression of *GAL*4 by binding with internal galactose (Gal), forming a *GAL*3-galactose complex, *g*_3_^c^, that inactivates *g*_80_ by binding to it and resulting in a complex *g*_80_^c^. In addition, the transporter *GAL*2 increases the amount of internal galactose, which stimulates the galactose network. We use the *GAL*1 mRNA level, *m_1_*, as the measureable output to control the system. According to sensitivity analysis, the interaction loops involving GAL4p dimerization (the dotted lines) can be eliminated from the original model to create a reduced model.

The *GAL* network in yeast, *Saccharomyces cerevisiae*, is one of the most studied gene networks, and hence is well suited to serve as a demonstration, benchmark system. Galactose utilization in the network can be broken up into four distinct stages based on proteins present in the cytoplasm. In the first stage *GAL*2p, the galactose transporter protein, allows galactose to enter into the cytoplasm [6]. Once internalized, *GAL*3p binds to the cytoplasmic galactose and is activated. This causes the galactose to bind and inhibit the action of *GAL*80p [7], [8]. With *GAL*80p bound, *GAL*4p is able to activate the transcription of *GAL*2, *GAL*3, and *GAL*80 genes. In the presence of glucose, *GAL*4p is also inhibited, which leads to inhibition of *GAL*1 expression [9]. Without accounting for the glucose network, the *GAL* system is composed of two positive feedback loops, *GAL*3p and *GAL*2p, and two negative feedback loops, *GAL*80p and *GAL*1p. The glucose network proteins bind upstream *GAL*1, *GAL*3, and *GAL*4, and suppress transcription through the action of Mig1. The glucose network also prevents the binding of *GAL*2p and external galactose and shuts down the galactose transportation. A mathematical model of the galactose network has previously been established by de Atauri *et al.* [10] and Ramsey *et al.* [11]. Bennett *et al.* combined a simplified glucose network with the galactose network model [12], and identified the model parameters using experimental data. A diagram of the simplified glucose and galactose networks used in this study is shown in **Figure 1**.

The key factor in designing a control system for a complex network that can be controlled externally is to design the system with measurable input(s) and output(s). For our study of the *GAL* network, the measurable inputs into the system are the concentrations of galactose and glucose. The most common mechanism currently available to monitor the output is a reporter to a gene of interest, normally green fluorescent protein (GFP), which allows researchers to measure the expression level of the gene. In the theoretical control model designed in this study, a GFP reporter coupled to *GAL*1 can be used to measure the output from the system. In practice, a spectrofluorometer or a microscope with appropriate excitation and emission filters, possibly coupled to a microfluidic device, could be used for measuring the GFP concentration [3]. It would then be possible to develop a control algorithm for the expression of *GAL*1 by tuning the concentration of galactose delivered externally to the cells. This model could be expanded to the control of other genes of interest (*GAL2*, *GAL*3, and *GAL*80) coupled to separate reporters.

Another challenge for control system design is the network scale and model complexity [13]. A gene network model is often constructed based on chemical kinetics with tens or hundreds of states and many parameters [12], [14]. The most successful model for the yeast cell cycle alone has 61 coupled ordinary differential equations and 141 parameters [15]. In such models, many important system characteristics, such as steady states, cannot be solved analytically and have to be estimated using numerical simulation. Advanced control mechanisms directly applied to these systems may waste considerable time on calculation of complicated, yet inessential nonlinear reaction terms. This complexity may lead to fragility of the controlled system, causing the system to collapse due to noise and parameter perturbation. The reduction of model complexity thus becomes extremely important for complex control system design and implementation. One effective tool for model reduction is parameter sensitivity analysis, which elucidates the dependence of system dynamics on the parameters. A small sensitivity measure for a parameter implies that the value of this parameter can be substituted for a wide range of values without altering the system dynamics. Some reaction terms can thus be deemed negligible and result in a reduced model. The control design based on the reduced model can be expected to have a similar performance to that of the original model. This paper uses the *GAL*1 mRNA level as a measureable output to be controlled (with the assumption that mRNA level is highly correlated with *GAL*1 translation) and investigates the sensitivity between the output as well as model parameters. The resulting reduced model based on sensitivity analysis successfully separates the galactose utilization network and the glucose network, and reduces the *GAL*4p subsystem and associated complexes. Furthermore, global sensitivity was also conducted to show how the system changes due to the simultaneous variation of all the parameters over a wide range of values. We have concluded that the global sensitivity analysis is consistent with the results of local sensitivity. In addition, the nonlinearity of the system is largely reduced, while maintaining a small deviation in the system dynamics. After system analysis, the original complex system was simplified to allow for the control system design. The goal of the control is to maintain one of the *GAL* mRNAs at a desired level.

In this paper, both a constant open-loop control and a proportional-output feedback closed-loop control are designed based on the reduced model. First, an analytic formula for the precise dosage of the external galactose is given, instead of manually tuning galactose by trial and error. Feedback control is introduced to improve the robustness of the controlled system and enhance the convergence rates. The simulation results show that both controls can achieve the control objective, *i.e.*, maintaining *GAL*1 mRNA at a desired level. Similar analytic galactose dosages can be achieved for all the other measurable outputs, such as *GAL*2, *GAL*3, and *GAL*80. The difference between the open-loop control of the system with constant control input, and feedback control with time-dependent parameters, is that the feedback control significantly shortens the time required to achieve the steady state. The feedback loop indirectly increases the degradation rate of the internal galactose so that the system more rapidly reaches balance. The feedback control also significantly reduces parameter sensitivity. Feedback control takes advantage of information about the system state to regulate output, and in doing so significantly decreases the local sensitivity of measurements to the system parameters. Hence feedback control can resist much larger parameter perturbations/uncertainties as compared to the constant open-loop control; that is, an open-loop control with constant control input.

## Methods

### Mathematical Model for Yeast GAL Network

The *GAL* network is a good starting point to demonstrate feedback and feed forward external controls for cellular systems. Though simple, it is well understood and is easily manageable to interpret experimental results. On the other hand, it is complex enough to test sophisticated control algorithms. As illustrated in Figure 1, a mathematical model for the *GAL* network has been proposed based on the interactions between proteins and internalized galactose. The figure shows a gene regulatory network for galactose utilization (redrawn from Bennett *et al.* (2008) [12]). The extracellular inputs are [*gal*]_e_ and [*glu*]_e_. The natural output of the network is a group of proteins. The *GAL*4 protein *g*_4_ binds to upstream activation sites and activates the regulatory genes in the galactose network. The *GAL*80 gene inhibits the inducing effects of *GAL*4 and thereby provides negative feedback in the system. *GAL*3 enhances expression of *GAL*4 by binding with internal galactose (Gal), forming a *GAL*3-galactose complex, *g*_3_^c^, that inactivates *g*_80_ by binding to it and resulting in a complex *g*_80_^c^. In addition, the transporter *GAL*2 increases the amount of internal galactose, which stimulates the galactose network. We use the *GAL*1 mRNA level, *m_1_*, as the measureable output to control the system. According to sensitivity analysis, the interaction loops involving GAL4p dimerization (the dotted lines) can be eliminated from the original model to create a reduced model. In the figure, *g_i_* is the number of the galactose network protein monomers (*i* = 1, 2, 3, 4, 80), *m_i_* is amount of mRNA (*i* = 1, 2, 3, 80), *g_id_* is the number of protein dimers (*i* = 4, 80), 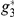 is the number of *GAL*3p proteins bound to galactose, 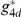 is the number of *GAL*4p dimers bound to Gal80p dimers, and 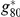 is the number of *GAL*80p proteins bound to the Gal3p-galactose complex.

A complete mathematical model of the above gene network, which we term the Original model, can be described with 22 mathematical equations [12]. The first five represent the mass-action kinetics of galactose protein monomers, including dimerization, and the interaction with the internal galactose:



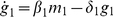
(1)

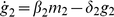
(2)




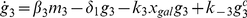
(3)

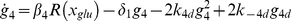
(4)

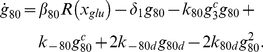
(5)Equations (6)–(9) describe mRNA kinetics accounting for the transcription and translation of the galactose genes as well as the degradation of mRNA



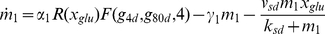
(6)

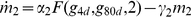
(7)

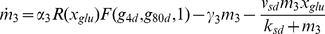
(8)

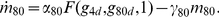
(9)Equations (10) – (14) represent the kinetics of protein dimers and the associated complexes:
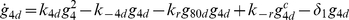
(10)

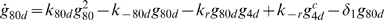
(11)

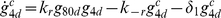
(12)

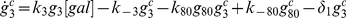
(13)

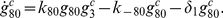
(14)The metabolic reactions and transport of galactose are represented by a single equation:



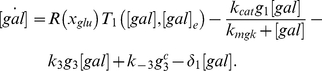
(15)Equations (16) – (18) describe a simplified glucose network, including the glucose-mediated enzymatic decay of *GAL1* and *GAL3* mRNA:



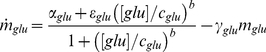
(16)

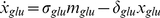
(17)

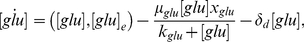
(18)where *m_glu_*, *x_glu_*, and [*glu*] are the amount of glucose network mRNA, associated protein, and cellular internal glucose, respectively. They compose a simplified glucose network. The inhibitory effect due to products of the glucose network that act on various processes of the galactose network is represented as follows,
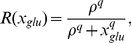
(19)where
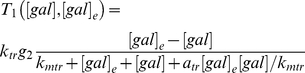
(20)and
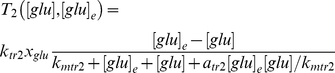
(21)are the transport rates of external galactose and external glucose into the cell, respectively. The cooperative fractional saturation function describing the number of upstream activation sites occupied on a promoter, assuming that *N* sites exist [10], is given by
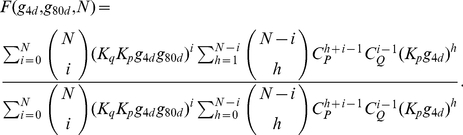
(22)The above mathematical model is mainly based on the biochemical reactions occurring throughout the network. This approach of modeling the intermediates in a pathway has been widely used in both chemistry and biology. The variables and parameters used for the model are defined and summarized in **Tables 1** and **2**.

**Table 1:** Model Variables.

Variable	Description	Initial value (molec.) [11]
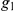 (molec./cell)	Gal1p	132.3
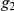 (molec./cell)	Gal2p	1156.7
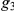 (molec./cell)	Gal3p	4341.2
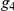 (molec./cell)	Gal4p	0.1563
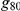 (molec./cell)	Gal80p	0.1138
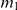 (molec./cell)	*GAL*1 mRNA	0.2647
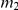 (molec./cell)	*GAL*2 mRNA	0.3305
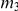 (molec./cell)	*GAL*3 mRNA	0.9044
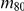 (molec./cell)	*GAL*80 mRNA	1.1871
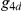 (molec./cell)	Gal4p dimer	308.92
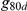 (molec./cell)	Gal80p dimer	157.229
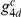 (molec./cell)	Gal4p dimer - Gal80p dimer complex	0
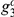 (molec./cell)	Gal3p – gal. complex	0
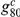 (molec./cell)	Gal80p – Gal3p – Gal. complex	0
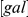 (molec./cell)	Internal galactose	0
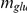 (molec./cell)	Glucose network mRNA	4000
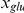 (molec./cell)	Glucose network proteins	15000
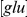 (molec./cell)	Internal glucose	150003
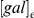 (molec.)	External galactose outside the cell	0– 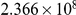
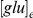 (molec.)	External glucose outside the cell	0– 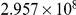

**Table 2:** Model Parameters.

Parameter	Description	Value [12]
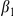 (min^−1^)	Translation rate of Gal1p *g*_1_	9.92
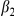 (min^−1^)	Translation rate of Gal2p *g*_2_	6.94
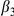 (min^−1^)	Translation rate of Gal3p *g*_3_	18.0
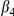 ((molec./cell)/min)	Max translation rate of Gal4p *g*_4_	0.86
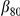 (min^−1^)	Translation rate of Gal80p *g*_80_	4.00
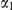 ((molec./cell)/min)	Max translation rate of *GAL*1	1.09
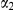 ((molec./cell)/min)	Max translation rate of *GAL*2	1.20
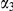 ((molec./cell)/min)	Max translation rate of *GAL*3	36.0
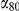 ((molec./cell)/min)	Max translation rate of *GAL*80	3.00
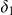 (min^−1^)	Deg. rate of gal. proteins and complexes	0.0033
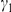 (min^−1^)	Deg. rate of *GAL*1 mRNA	0.036
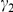 (min^−1^)	Deg. rate of *GAL*2 mRNA	0.026
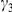 (min^−1^)	Deg. rate of *GAL*3 mRNA	0.036
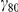 (min^−1^)	Deg. rate of *GAL*80 mRNA	0.036
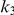 ((molec./cell)/min)	Binding rate of 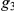 to 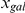	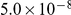
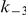 (min^−1^)	Dissociation rate of 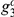	890
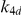 ((molec./cell)/min)	Binding rate of 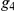 to 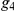	0.1
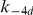 (min^−1^)	Dissociation rate of 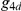	1.0
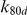 ((molec./cell)/min)	Binding rate of 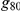 to 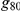	0.10
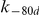 (min^−1^)	Dissociation rate of 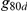	170
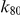 ((molec./cell)/min)	Binding rate of 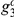 to 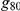	0.10
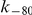 (min^−1^)	Dissociation rate of 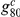	0.03
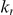 ((molec./cell)/min)	Binding rate of 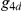 to 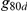	0.10
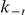 (min^−1^)	Dissociation rate of 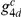	1.80
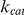 (min^−1^)	Galactose metabolism rate	3350
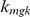 (molec./cell)	Galactose metabolism constant	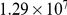
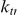 (min^−1^)	Galactose transport rate	4350
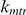 (molec./cell)	Galactose transport constant	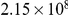
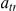 (unitless)	Galactose interactive constant	10.0
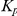 (cell/molec.)	Equilibrium constant of 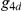 binding to UAS	0.091
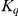 (cell/molec.)	Equilibrium constant of 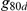 binding to 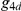	0.0556
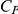 (unitless)	Cooperative binding constant of 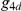 to UAS	1
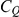 (unitless)	Cooperative binding constant of 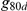 to 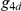 -UAS complexes	30
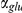 ((molec./cell)/min)	Basal transcription rate of glucose DNA	215
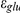 ((molec./cell)/min)	Activated transcription rate of glucose DNA	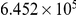
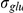 (min^−1^)	Translation rate of glucose proteins	0.4
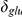 (min^−1^)	Degradation rate of glucose proteins	0.1
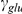 (min^−1^)	Degradation rate of glucose mRNA	0.0633
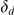 (min^−1^)	Dilution rate of glucose	0.0033
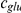 (molec./cell)	Hill constant for glucose induction	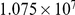
b (unitless)	Hill coefficient for glucose induction	1.8
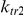 (min^−1^)	Glucose transport rate	4350
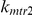 (molec./cell)	Glucose transport constant	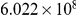
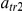 (unitless)	Glucose interactive constant	1.0
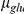 (min^−1^)	Glucose transport rate	5350
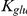 (molec./cell)	Glucose transport constant	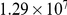
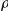 (molec./cell)	Hill constant for gal. repression	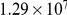
*q* (unitless)	Hill coefficient for gal. repression	0.8
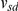 (min^−1^)	Glucose induced mRNA degradation rate	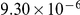
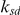 (molec./cell)	Glucose induced mRNA degradation constant	30

Our goal for galactose network control is to regulate the *GAL* family mRNA level by manipulating galactose and glucose concentrations. In this paper, we select *GAL*1 as the gene of interest. The mRNA level of this target gene can be measured experimentally using a GFP conjugated to *GAL*1, which can be measured for intact cells, or by means of a microarray or other assay that requires lysing a small fraction of the cells under observation. In practice, other reporter genes may be used to monitor *GAL*1 expression, for example, the red fluorescent protein from the gene dsRed [16]. In addition, it is notable that glucose suppresses all *GAL* genes [17]. External glucose must be kept at a very low level, *i.e*., 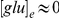, or the *GAL* network will be inhibited. Once no external glucose is supplied, the glucose network will soon reach its steady state, which means one can focus on the galactose network.

From a control engineering perspective, one key step is to identify inputs (control) and outputs (preferably measurable), so that the system can be put into a control framework for discussion. 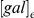 and 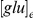, external galactose and glucose levels, are the control variables in this study, while the mRNA levels of *GAL*1, *GAL*2, *GAL*3, and *GAL*80, *m*_1_, *m*_2_, *m*_3_, *m*_80_, are measurable variables. The goal of this research was to design a time-course of 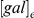 such that one of the mRNAs *m*_1_, *m*_2_, *m*_3_, *m*_80_ is maintained at a desired level.

While there are several methods that might be suitable for culturing yeast under external control, microfluidic devices would be ideal for experimental implementation of the cellular control system [18]. Fluorescence microscopy could be used to quantify, in real-time, the levels of GFP-labeled *Gal*1p from a small, synchronized population of *S. cerevisiae,* with the output of a camera or photomultiplier tube serving as the sensor measurement to the control system. Ion mobility-mass spectrometry could also be used to monitor the secreted metabolites and signaling molecules in real-time and intracellular species after cell lysis [19]. Microfluidic valves could control the concentrations of various chemicals and serve as the control system outputs.

In the next section, we will conduct a sensitivity analysis to simplify this subsystem. As shown in **Figure 1**, the interaction loops involving *GAL*4p dimerization can be removed. The reduced model lowers the complexity to design the associated controller, allowing analytical implementation of a feasible design. We will show later that the reduced model may cause bounded deviation from the original model in transient response; however, it results in insignificant deviation from the steady states.

### Model Reduction

Given the steady states of the glucose utilization network, the original model still has 35 parameters. The large number of parameters and significant nonlinearities make it difficult to design an effective controller. As with any biological system, the number of parameters involved in the induction of a specific pathway could be large, with physical, chemical, and biological parameters all affecting the system. In terms of a modeling-based representation of biological systems, the goal of our effort is to determine the parameters that most affect the induction of the system.

A simple example of this concept can be observed in glycolysis. Through the complex intermediates and interactions within this pathway, three regulatory steps are often considered, biologically, the most important rate-limiting steps in the pathway. These irreversible steps, phosphorylation of glucose, phosphorylation of fructose-6-phosphate, and transfer of phosphate to phosphoenolpyruvate and then to ADP, would be the most important control points. The proteins involved in these steps, as well as the mRNA that generates these proteins, are crucial to the overall process. Investigating the influence of the parameters on the control target is an effective tool to reduce the model complexity and assist control system design. In order to reduce the model, we should know whether the output significantly changes under a small change of the parameters, as some parameters may not be as effective as others. By understanding how small changes in parameters affect the overall system, we can determine the most important parameters involved in generating the desired output. In the case of *GAL*1 mRNA, a small change in the value of the *GAL*4p parameter will have a large effect on *GAL*1 mRNA expression, indicating that this parameter significantly influences the uncertainties of *GAL*1 mRNA expression. Thus, this parameter is crucial to control the system. Small changes in a parameter that was not as closely linked to *GAL*1 mRNA expression specifically may not have a large effect on the expression. In fact, most biological systems may be sensitive to certain parameters over a large range. If the sensitivity of a parameter is small compared to other parameters, the parameter could be arbitrarily chosen from a wide range of values without altering the dynamic performance of the system. This practice has been used successfully in control systems engineering, including chemical process control, robotics control, aerospace control, etc.

For the above model, we will first conduct local sensitivity analysis that will quantify the change of the system states due to constant perturbation of a parameter at time zero. Consider a general form of nonlinear ordinary differential equations:



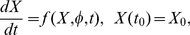
(23)where *X* is the state vector with *n* components, and 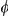 is the parameter vector with *m* components. The initial condition of the above equations is set to *x*_0_. Define the sensitivity coefficients of the state *x_i_* with respect to parameter 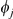, *j* = 1, …, *m*, by



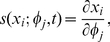
(24)and then the state's sensitivity trajectory can be described by the following:



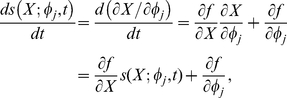
(25)where the state vector is denoted by 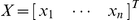. The initial values of the sensitivity equations can be obtained by



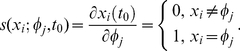
(26)


In order to avoid scale differences in the parameters, the sensitivity coefficients are normalized as follows,



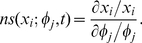
(27)


The normalized sensitivity coefficient 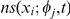 means that a 1 percent change of state *x_i_* results in a 1 percent change of the parameter 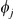 over the time course *t*. The sensitivity trajectory 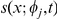 can be calculated numerically by equations (25–26). The importance of the parameters can thus be distinguished by the magnitude of the normalized sensitivity coefficients in the time-course of the system following the constant perturbation at time zero.

**Figure 2:**
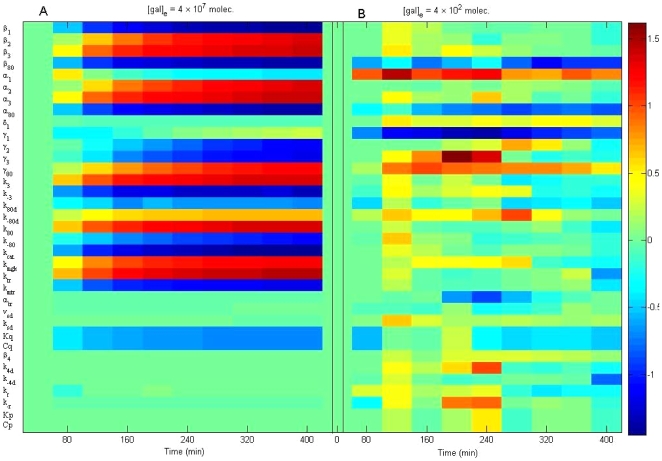
Normalized parameter sensitivity trajectory with respect to time. The heat map shows the normalized sensitivity of *GAL*1 mRNA concentration (*m*_1_) towards change in all 35 parameters. Panel (A): Sensitivities when 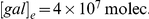 Panel (B): Sensitivities when 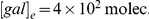 The sensitivities of the parameters 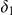, 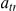, 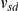, 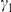, 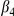, 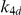, 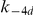, 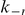, 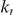, 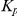, 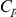 and 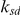 are much smaller than those of the other parameters (approximately zero, green color). In general, when the external galactose 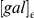 is higher, the parameters are more sensitive, besides α_1_ and γ_1_.

The normalized sensitivity coefficients for the original model are shown in **Figure 2**, where each small block describes a normalized sensitivity for a certain parameter at a certain time. In **Figure 2A**, the galactose input is kept at a constant, 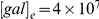 (molec.) with the measured system output represented by *m*_1_. It is easy to observe that the external galactose input is positively correlated to the output of *m*_1_. The glucose network remains in the steady state when the internal glucose is emptied and there is no external glucose supply. The steady state of 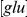  = 13586 (molec./cell) and 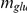 = 3397 (molec./cell) can be determined by solving 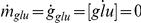. The function 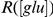 in equation (19) was found to equal 0.9959. Thus, the glucose network can be separated from the galactose network. This is in agreement with experimental data, where a lack of glucose as a carbon source and the presence of galactose will induce the *GAL* network. Based on the sensitivity analysis of *m*_1_ with respect to all the parameters (**Table 2**), it becomes clear which parameters can be omitted from the system. The heat map in **Figure 2** shows the normalized sensitivity of *GAL*1 mRNA concentration (*m*_1_) after changes in all 35 parameters. The **Figure 2A** shows the sensitivities when 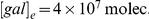 The **Figure 2B** represents the sensitivities when 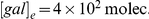 The sensitivities of the parameters 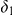, 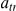, 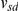, 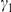, 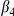, 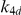, 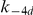, 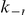, 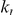, 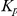, 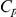 and 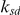 are much smaller than those of the other parameters (near zero, green color). In general, when the external galactose 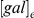 is higher, the parameters are more sensitive, with the exception of α_1_ and γ_1_.

However, it would be careless to omit these parameters without further investigation. For example, the degradation rate 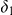 is important for achieving the steady states of the original model, so it must be retained. 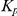 and 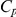 come from the cooperative fractional saturation function 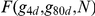. Both 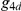 and 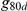 in the function contribute to the activation of mRNAs *m*_1_, *m*_2_, *m*_3_, and *m*_80_. However, the contributions may not be equal. If we define 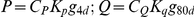, then we have
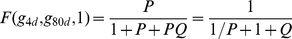
(28)When 1 + Q >>1/*P*, the above equation (28) can be approximated by
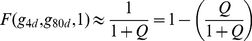
(29)


From (29), we observed that the output *m*_1_ is positively correlated to the external galactose input. The sensitivity analysis has told us that 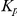 and 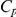 can be set to be large enough to satisfy 1 + Q >>1/*P*. Thus, only 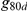 contributes to the cooperative fractional saturation function. Similarly, the other two cooperative functions can be simplified as follows,
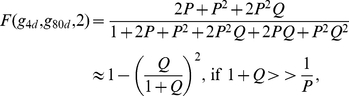
(30)and
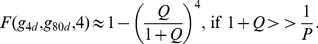
(31)


**Figure 3:**
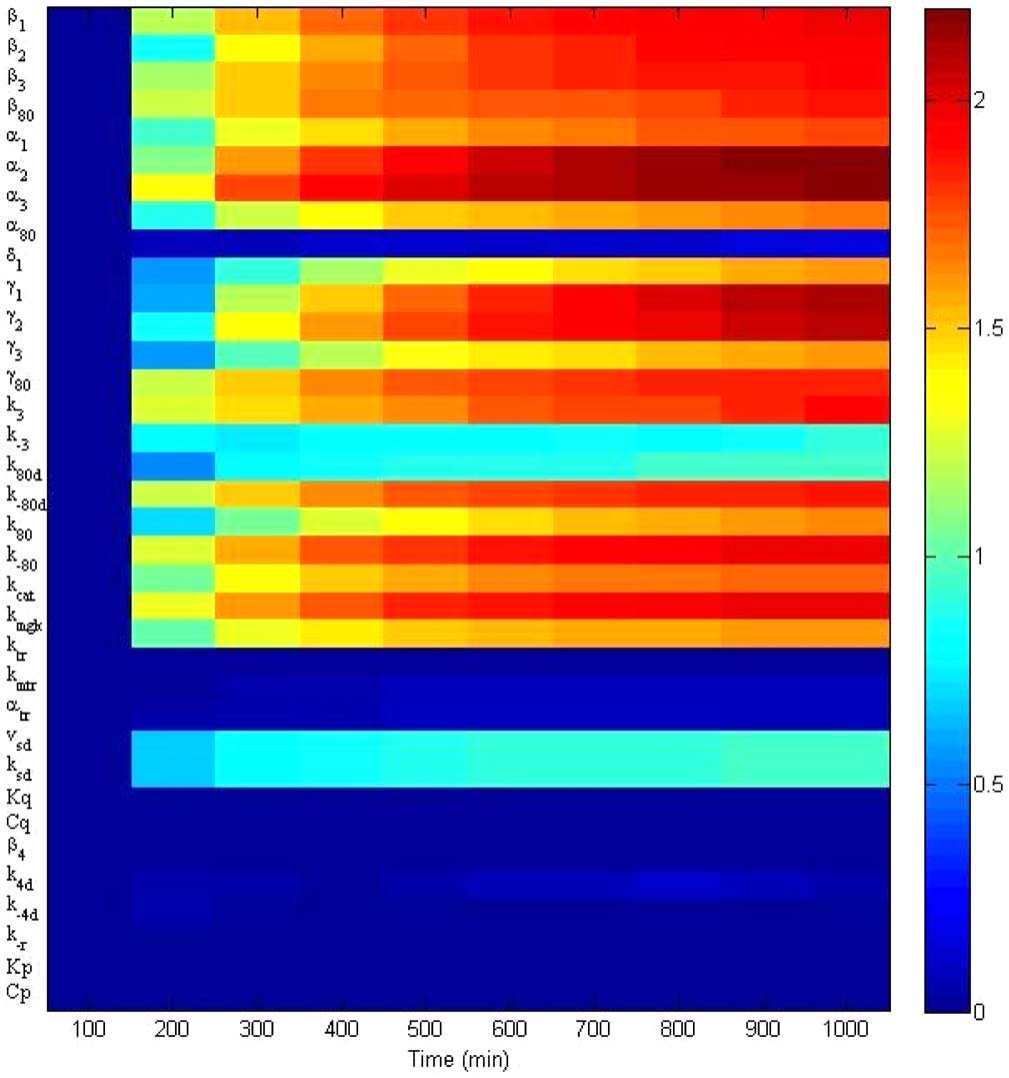
Local sensitivity analysis at the steady states of four *GAL* mRNAs with respect to all model parameters. The heat map denotes the average absolute value of the normalized sensitivity coefficients of four *GAL* mRNA across 1000 minutes. The parameters 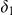, 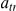, 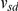, 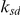, 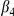, 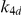, 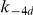, 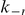, 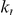, 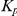, and 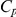 are smaller than 0.25, while the others are larger than 1. This implies the reaction terms associated with insensitive parameters can be omitted no matter which mRNA is chosen as the control target. One can conclude not only that the control model targeting *GAL*1 mRNA need not include the terms associated with the above parameters, but also that control strategies targeting all four *GAL* mRNAs can suppress the same terms. The reduced model can thus be used for the control design targeting any of the four *GAL* mRNAs.

**Figure 4:**
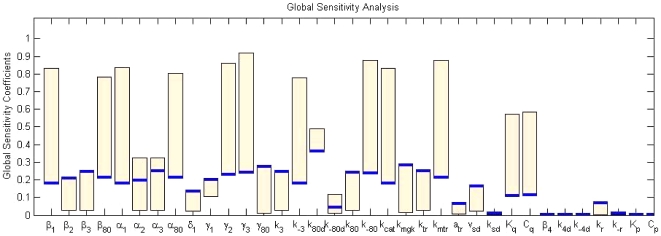
Global sensitivity analysis at the steady states of four *GAL* mRNAs with respect to all model parameters under constant control 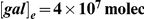
 **.** The bars denote the range of the sensitivity coefficients of four *GAL* mRNAs with respect to each parameter. The blue solid line stands for the global sensitivity coefficients for *GAL*1 mRNA.

Therefore, the cooperative fractional saturation effect of *g*_4d_ and *g*_80d_ can be approximated by the contribution from 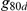 alone. This can also be explained by the sensitivity analysis in **Figures 1**, **3**, and **4**, which shows that 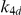, 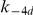, 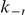 as well as 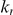 are less “essential” to the system output. Thus, we can set 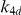, 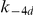, 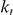, and 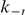 to be zero, since they are small numbers and have small sensitivity coefficients. This results in a removal of the loops involving the *GAL*4p dimer, 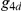, and the *GAL*4p/*GAL*80p dimer complex, 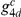. As shown in **Figure 1**, the subsystem (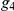, 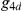, 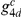 and 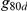) can be simplified as 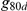 only, because the rest of the subsystems will not affect the steady state of the output. The goal of the proposed control is to maintain a level of mRNA expression at some predetermined level. The model reduction will thus have little influence on the control performance in terms of output deviation after a long enough time. The contributions from the glucose network to *m*_1,_ and *m*_3_, are small when no glucose exists, which is necessary to induce the *GAL* gene network, as explained earlier. This is consistent with the small sensitivity coefficients with respect to 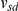 and 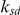. We thus set 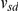 equal to zero and 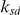 equal to a large number. Also, small sensitivity of the coefficient with respect to 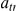 leads to a slight model reduction on 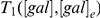 by setting 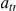  = 0.

Thus, a Reduced Order Model can be achieved based on the analysis above. The Reduced Order Model in a state space form can thus be described as follows:
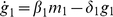
(32)

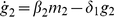
(33)

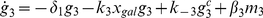
(34)

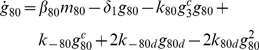
(35)

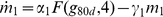
(36)

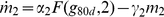
(37)

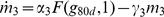
(38)

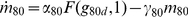
(39)

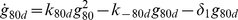
(40)

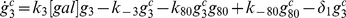
(41)

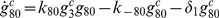
(42)

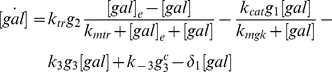
(43)where



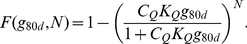
(44)All parameters are summarized in **Table 2**.

**Figure 5:**
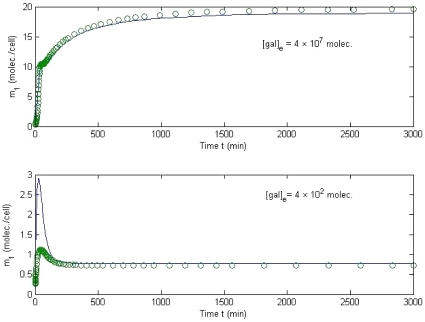
Comparison of original model and reduced model. The difference between the two models occurs in the transition process before the first 200 min, while the difference decreases significantly in the steady state. When 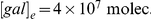 and 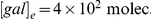 are applied to both models, the steady-state deviation of the output *m*_1_ (molec./cell) is less than 1 percent. However, the deviation of the transition process between the two models with the smaller external galactose is significant. This result is consistent with the sensitivity measure in Figure 3. The solid line is for *m*_1_ concentration in the original model, while the open symbol is for the reduced model.

The comparison of the outputs of the Original Model and the Reduced Model is shown in **Figure 5**. The differences between the two models occur in the transition process before the first 200 min, while the difference decreases significantly in the steady state. According to the experimental results from [11], [12], the galactose network takes 4–7 hours (240–420 minutes) to reach a steady state. Our simulation results in **Figure 3** are consistent with the experimental data. When the external galactose level is set at 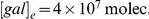 or 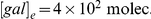, the steady-state deviation of *GAL*1 mRNA, *m*_1_ (molec./cell) is similar between the full and reduced models. However, the deviation of the transition process, the time before achieving the steady state, between the two models with the smaller external galactose level is significant. This result is consistent with the sensitivity measure in **Figure 3**.

Biologically, the reduced model varies from the complex model only within the first 200 minutes of expression for relatively low galactose levels, 400 molecules. The steady-state levels of *GAL*1 mRNA expression after the first 200 min for both galactose levels tested were the same in both models (**Figure 5**). In this way, the reduced model can be used to explain the expression of *GAL*1 mRNA, once the expression level has reached a steady state. Before the steady state is reached, only a complex model can completely model the effects of *GAL*1 mRNA expression from the introduction of low levels of galactose. A similar technique can be applied if the control target changes to any of the other mRNAs or proteins. This paper exemplifies *GAL*1 mRNA as a primary control target, but the applications of the method are not limited to a certain mRNA. Choosing *GAL*2 or *GAL*3, as well as *GAL*80, will produce very similar reduced models; however, using *GAL*4 as a control target will obviously yield a different reduced model because of the simplification in the *GAL*1 output system obtained by the removal of the *GAL*4p subsystem. A reduced model for *GAL*4 would have to include the *GAL*4p subsystem, and thus a much different reduced model would be generated.

**Figure 3** describes the local sensitivity analysis at the steady states of four *GAL* mRNAs with respect to all model parameters. The bars denote the range of the sensitivity coefficients of four *GAL* mRNAs with respect to each parameter. The parameters 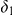, 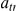, 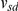, 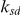, 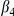, 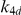, 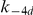, 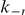, 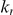, 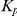, and 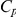 are smaller than 0.25, while the others are greater than one. This implies that the reaction terms associated with the insensitive parameters can be omitted no matter which mRNA is chosen as the control target. The design of a control model to control steady-state expression of *GAL*1, *GAL*2, *GAL*3, and *GAL*80 mRNA (*m*_1_, *m*_2_, *m*_3_ and *m*_80_) is described in the following section.

The local sensitivity analysis describes the change of system dynamics at time *t* after a step-wise perturbation to a parameter occurs at time zero. One might wonder what the effect would be when all parameters are perturbed simultaneously. Global sensitivity analysis investigates the effect of simultaneous large variations of all parameters on the states. The common methods to test global sensitivity include the Fourier amplitude sensitivity test (FAST) [20], extended FAST [21], Sobol's method [22], the partial rank correlation coefficient (PRCC) [23], as well as the weighted average local sensitivity method [24].

FAST is one of the classical methods to determine global sensitivity. Assuming that the time dependence of each uncertain parameter 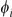 is associated with a frequency 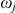, we can describe that parameter by a transformation function of a sinusoid, *i.e.*,



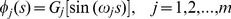
(45)for all *m* parameters, where *s* is the time.

The first order global sensitivity of *x_i_* with respect to the variation of the parameter 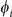 can be defined as the ratio of the variance of 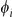 to the total variance



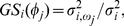
(46)where the total variance is
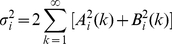
(47)and the variance of 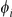 becomes



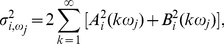
(48)with



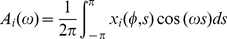
(49)




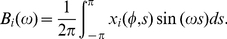
(50)


Although the above local sensitivity analysis pointed out some candidate parameters and their associated terms that may be removed, it only guarantees the scenario when the parameters are tuned one by one. When the parameters are simultaneously adjusted, some may become sensitive. Thus, we must also apply global sensitivity analysis to rule out corresponding parameters.

As shown in Figure 4, the bars denote the range of the sensitivity coefficients of four *GAL* mRNAs with respect to each parameter under constant control 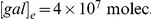 The blue solid line stands for the global sensitivity coefficients for *GAL*1 mRNA. In particular, 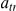, 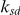, 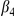, 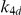, 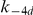, 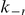, 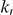, 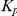, and 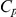 are nearly zero. The maximum of the coefficients 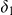, 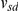 and 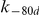 is smaller than 0.2, while that of the others is larger than 0.2, consistent with the local sensitivity coefficients in Figure 3. This implies that the reaction terms associated with insensitive parameters can be omitted no matter which mRNA is chosen as the control target. Thus, both local and global sensitivity analyses indicate that the reduced model, equations (32–43), can be used for regulating all four *GAL* mRNAs.

### Control Design

As discussed earlier, any of the four *GAL* mRNAs can be chosen as the control target. For demonstration purposes, we have chosen to control *GAL*1 mRNA (*m*_1_), *i.e.*, essentially maintain the *GAL*1 mRNA at a desired level 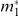, *i.e*.,



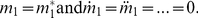
(51)In order to keep equation (51) true, the steady state of the system becomes



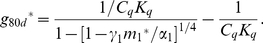
(52)


As a test case for this model, we will demonstrate the control of *GAL*1 mRNA expression by controlling the external level of galactose. It is important to note that any gene or protein represented in the reduced model can be controlled in a similar manner. Following this same pattern, recombinant genes regulated by *GAL*1 could be controlled in the same manner.

Similarly, we can find the steady states for 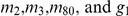,



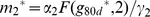
(53)




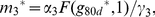
(54)




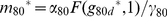
(55)




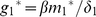
(56)




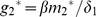
(57)




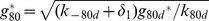
(58)




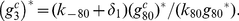
(59)


Observing the galactose network, we can obtain two auxiliary equations:



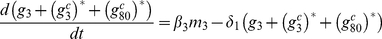
(60)




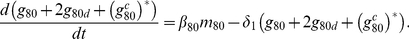
(61)


Equation (60) indicates that the total mass of 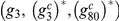, is up-regulated by *m*_3_. Biologically, this is a simple concept to understand, as expression of *GAL*3 mRNA, *m*_3_, is necessary for the generation of *GAL*3p, 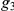, and the Gal3p dimer, 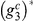, in a dose-dependent manner. Similarly, the *GAL*80p/*GAL*3p dimer complex is dependent on the expression of *GAL*3p. Equation (61) indicates that the total mass of 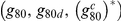, is up-regulated by *m*_80_, in a manner similar to that described above. Both of these equations show the interplay between the biological mechanisms responsible for their creation.

Through equations (60) and (61) we can determine the remaining steady states 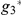, 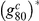 and 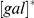 using the auxiliary equations



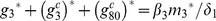
(62)




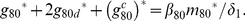
(63)


Thus,



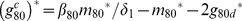
(64)




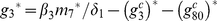
(65)




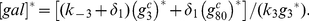
(66)


Given our desire to enforce the control condition of 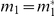, we need to control the external galactose, 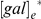
_,_ which at steady state can be calculated as follows,



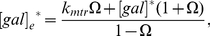
(67)where 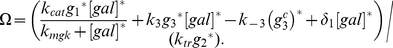


Similarly, if the control objective is to regulate *GAL*2 mRNA, 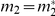, then



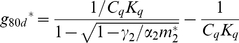
(68)




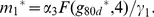
(69)


The rest of the terms can be achieved using the same equations (54)–(67). Because this system has only a single steady state, one can always find a nominal control value for external galactose 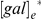 to maintain any control objective at a desired level. In other words, we can always compute the necessary amount of galactose offline in order to maintain *GAL*1 mRNA or any other factor, such as *GAL*2 mRNA or *GAL*3p, at a level of interest.

Thus, offline, we can calculate an open-loop constant control, which is the simplest type of controller that does not take into consideration state information that is fed back into the system for control purposes.



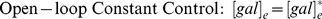
(70)for any of the targeting mRNA, *m*_1_, *m*_2_, *m*_3_, and *m*_80_, if these mRNAs are not measurable in real-time. However, if they are measurable, one can improve the control algorithm by introducing a closed-loop feedback control. A simple proportional output feedback control for *m*_1_ can be described as



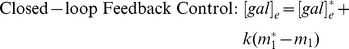
(71)where the feedback gain 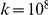. The gain *k* directly affects the convergence rate of the controlled GAL network. However, the external galactose amount should not exceed the maximum concentration of galactose. Thus, the control dosage should be bounded by



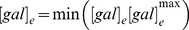
(72)Of course, one can use a proportional-integral-derivative (PID) control in practice. Integral control can adjust the static error, but too much integral gain can reduce the stabilization of the system. Derivative control can adjust the convergence rates, however, it is too sensitive to high frequency noise. For pursuing different requirements, many more complicated control methods can be applied, such as optimal control for minimizing the galactose dosage and robust control for reducing the uncertainty, among others. Because the control objective of regulating the mRNA concentration to a desired value, is a relatively simple task, without strict requirements for reaction time and galactose dosage, a simple proportional control is adequate to accomplish the control goal.

## Results

### Control Stability Analysis

Next, we will prove the stability of this equilibrium because the equilibrium is a unique steady state. If it is stable, then the state will tend to this steady state given the nominal control. The control objective *m*_1_ will be maintained at the desired level 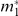. Biologically, this system is stable based on experimental results [12]. The following is a theoretical proof based on the mathematical model.

The Jacobian matrix at this equilibrium is



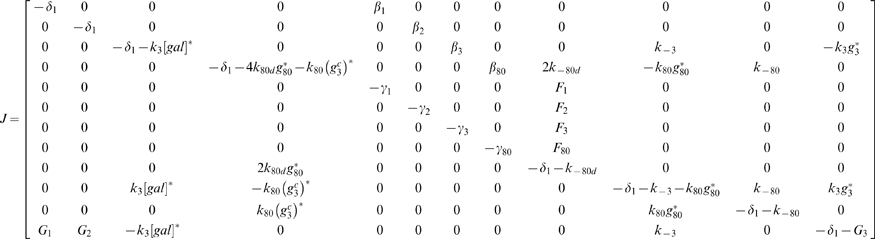
(73)


where



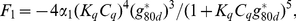
(74)




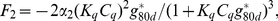
(75)




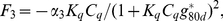
(76)




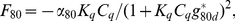
(77)




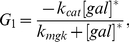
(78)




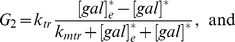
(79)




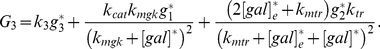
(80)


If 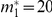, one finds the eigenvalues of the Jacobian matrix are [−895.99, −194.68, −12.54, −12.04, −0.0295, −0.0387+ 0.0024*i*, −0.0387− 0.0024*i*, −0.0360, −0.0015, −0.0033, −0.0033, −0.0033]. A standard stability analysis of the dynamic systems finds that the equilibrium is asymptotically stable. Thus the system will tend to the steady state asymptotically.

Because most of the eigenvalues are small in magnitude, it implies that the convergence of this controlled system will take a long time. Biologically, after we set the external galactose level to a constant value, the yeast galactose utilization network will slowly achieve balance (a steady state) and the *GAL*1 mRNA level will gradually reach and maintain the desired level. At this steady-state level, there will not be oscillations in the mRNA or protein levels associated with this system.

In order to change the eigenvalues to negative, we follow a common practice in control systems engineering and introduce a linear output feedback control



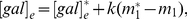
(81)where 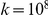. 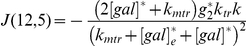. The eigenvalues of the Jacobian matrix indicate how fast the control can reach a steady state. In general, the more negative, the faster. In the above control, the eigenvalues of the Jacobian matrix (73) move far away from the y axis: [−895.99, −194.67, −16.40, −5.33, −2.91, −0.026, −0.0361, −0.0360, −0.0033, −0.0033, −0.0033]. This implies that the system will converge faster under the linear feedback control. Increasing the value of *k* will lead to faster convergence. However, the growth of the convergence rate is limited due to the maximum concentration of galactose. From **Figure 1**, we can find that the internalized galactose level increases with rising external galactose levels, and the internalized galactose level enhances *GAL* 4, which in turn promotes *GAL*1 mRNA expression. Increased *GAL*1 mRNA expression will also increase the degradation rate of the internalized galactose, thus the internalized galactose will reach a steady state faster. In essence, the introduction of the feedback control loop actually increases the external galactose level, but dynamically adjusts the amount such that the *GAL*1 mRNA can still reach the desired level. In this process, the degradation rate of the internalized galactose is largely enhanced, consistent with a significant increase of the magnitude of several negative eigenvalues.

**Figure 6:**
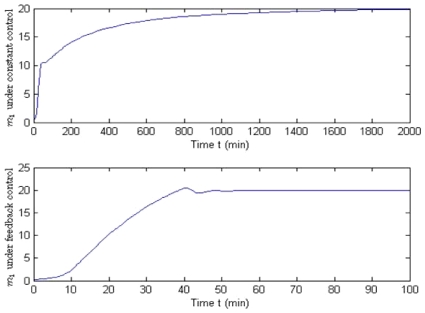
Comparison between constant and feedback control for the Reduced Order Model. It takes more than 1500 min for *GAL*1 mRNA, *m*_1_ to reach the desired level of 20 (molec./cell) using the constant control (top); however, only 40 min are required for the feedback control (bottom). According to the simulation, feedback control is much more effective at bringing the yeast system into a steady-state level of mRNA expression.

We set the desired level of *GAL*1 mRNA as *m*_1_ = 20. From equation (67), the nominal control values for [*gal*]_e_* can be obtained as 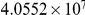 molec./cell. Application of the nominal control should cause the system to slowly reach a stable equilibrium. As shown in **Figure 6**, it takes more than 1500 min for *m*_1_ to reach the desired level. From **Figure 5**, we can see that achieving the desired high *GAL*1 mRNA level usually demands a much longer time than is required for low *GAL*1 mRNA levels. The metabolic reactions involved in promoting mRNA expression and subsequent translation into proteins require a lengthy time. Multiple positive and negative feedback loops inside the network actually prevent mRNAs and the associated proteins from rapid changes due to external stimuli.

**Figure 7:**
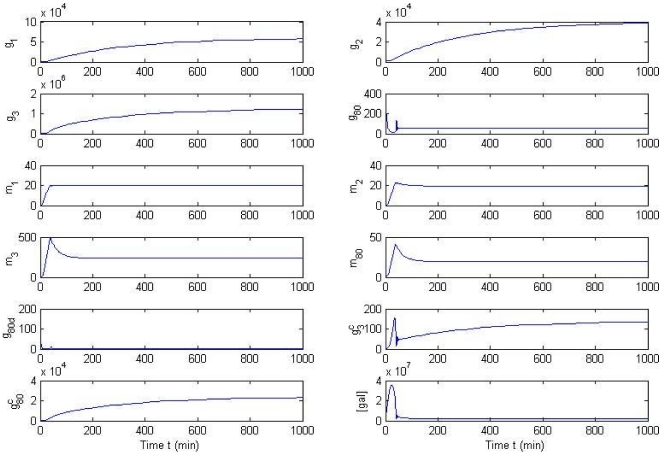
Effect of protein concentration on mRNA expression by feedback control on the *GAL* network. All states (unit: molec./cell) converge to a stable steady state, while *g*_2_, 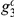 and 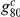 have slower convergence rates than the other states. The transient process of states 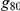 and 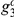 is listed in the small windows above. The overshoot of the states is kept in a reasonable range. No severe fluctuations or oscillations are found in the *GAL* network under feedback control.

The effects of protein concentration profiles by feedback control on *GAL*1 mRNA expression on the *GAL* network are illustrated in **Figure 7**. All states (unit: molec./cell) converge to a stable steady state, while *g*_2_, 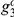 and 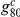 have slower convergence rates than the other states. It is not surprising to see that all concentrations tend to a steady state, since the system has a unique stable steady state. But the transient response of the galactose system is equally important, because a large fluctuation is not desired in a reliable bioreactor. The figure shows that the system does not have large overshoots and is thus safe for implementation in practice.

**Figure 8:**
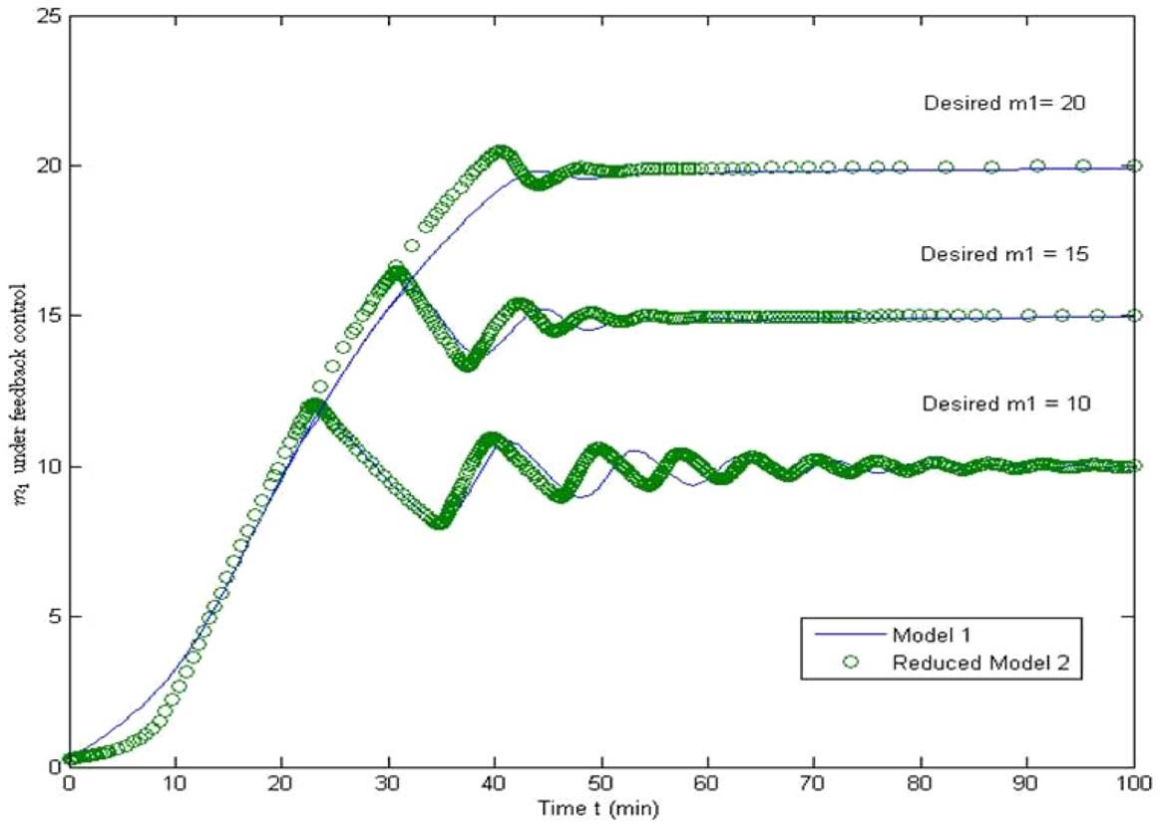
Comparison of referenced tracking between the original model and the reduced model using feedback control. Under the feedback control, the tracking performance of the original model and the reduced model is similar. Thus, the control design based on the reduced model can be applied to the original model directly.

**Figure 8** compares the set point regulation performance of the feedback control between the original model and the reduced model using feedback control. Setting three desired levels of *m*_1_ as 10, 15, and 20, feedback control based on both the original model (solid) and the reduced model (open) can be maintained at the desired levels within a similar time frame. The reduced model-based feedback control, however, leads to slightly larger magnitudes of oscillations at the transient response. The difference is small enough not to result in any severe fluctuation in the biological system. Thus, the control design based on the reduced model can be applied to the original model directly.

### Sensitivity Analysis

The steady-state sensitivity describes the change of the steady state given a small perturbation of a certain parameter [25]. It can be given by the first-order partial derivative of a state with respect to the parameter. Using the finite difference method, the normalized steady-state sensitivity (NSSS) coefficient for the steady state of state *x*_i_ with respect to the parameter 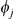 can be approximated by



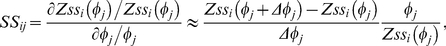
(82)where 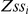 is the steady state of the state *x_i_*.

**Figure 9:**
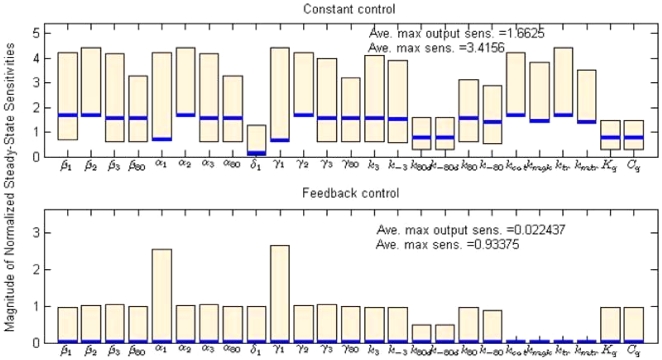
Comparison of normalized steady-state sensitivities (NSSS) between constant and feedback control. The NSSS of the feedback control case has significantly smaller magnitude in terms of average maximal sensitivities for all states (0.93 v.s. 3.42) and the output state (0.02 v.s. 1.66). The bars describe the maximum and minimum values of NSSS of the states with respect to each parameter. The solid lines denote the output NSSS on each parameter.

A small magnitude of NSSS of state *x_i_* with respect to the parameter 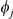 implies that the parameter uncertainty of 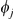 has no significant influence on the state *x_i_* locally. In contrast, a large NSSS implies serious influence by the parameter uncertainty. It is necessary to emphasize “locally” because the steady-state sensitivity is a local sensitivity measurement that perturbs one parameter and fixes the other parameters. As shown in **Figure 9**, we calculated the NSSS of all states with respect to the output *m*_1_ for both constant control and the feedback control algorithm. The NSSS of the feedback control case has significantly smaller magnitude in terms of average maximum sensitivities for all states (0.93 v.s. 3.42) and output state (0.02 v.s. 1.66). Especially for the output state, the magnitude of NSSS was reduced 100-fold. This implies that the parameter uncertainty does not affect the steady state of the output. The feedback control law (81) can always drive the output to the reference level subject to uncertainties and perturbation in the parameters locally.

Biologically, we can conclude from the above data that the feedback control is less influenced by disturbances within the system, as shown in **Figure 9** **.** For mRNA expression, this means that a feedback control prevents the system from being overly sensitive to any specific parameter. The perturbations or parameter uncertainty in any parameter will not cause a significant deviation to the steady mRNA level. This is necessary for complex biological systems, where parameter uncertainty and perturbations widely exist. If the system was overly sensitive over some parameters, then it would be difficult to maintain the mRNA at a desired level. A series of checks and balances are always present to prevent the system from fluctuating out of control.

## Discussion

This paper employs control theory and a systems engineering approach to demonstrate the regulation *in silico* of the budding yeast, *Saccharomyces cerevisiae*, into a desired gene expression and/or metabolic activity pattern. The *GAL* network is used as an example to demonstrate the effectiveness of a systems approach for complex cellular system control. We demonstrate that although the structure and computational models are complicated, it is possible to use a “simple” control algorithm to manipulate the system. According to the local parameter sensitivity analysis, some terms can be eliminated from the model without adversely affecting its long-term behavior, and the control design based on the reduced model can still be applied to the original model. Both constant control and feedback control laws have been designed for the galactose network. To evaluate the control performance, we conducted local steady-state sensitivity and global sensitivity analysis. The results accordingly imply that feedback control significantly suppresses the parameter uncertainties. This sheds light on the potential to provide novel control components to eukaryotic transcriptional regulation networks, as well as other gene regulatory networks.

The approach we propose can not only be used to control the yeast *GAL* network, but the fundamental principles can be applied to a wide range of biological network control. Complex networks involving cell-cycle controls, DNA repair, and other genes of interest can be controlled in a similar manner, given an effective means of monitoring the output of the system. The most attractive application is the control of bioreactors. If done properly, the ability to yield a large amount of the target in a relatively short period of time, while minimizing the effects of toxic levels of gene expression products can be achieved. If these systems are tightly controlled at the cellular level, proteins of interest, such as immunogenic proteins used in the production of vaccines, restriction enzymes, biopharmaceuticals, biochemicals can be tightly regulated. This would allow for a cost-effective means of producing a large variety of products for research and commercial use. Applications to other systems include bacterial expression systems and even mammalian cell systems, based on the effectiveness of the bioreactor and the access to the requisite control variables. Of course, other physical and chemical parameters would be necessary to completely control a bioreactor-based system, including pH, temperature, and nutrient conditions. The major difference between these and the above approach is that the proposed approach focuses on cellular system control, which is still a widely open field. Our future research will implement this control system with a fully established microfluidic device to monitor the real-time expression of GAL-GFP fusion proteins. By validating the above control in an experimental environment we will demonstrate the usefulness and validity of our approach.

## References

[1] Bhalla US, Iyengar R (1999). Emergent properties of networks of biological signaling pathways.. Science.

[2] Gardner TS, Cantor CR, Collins JJ (2000). Construction of a genetic toggle switch in Escherichia coli.. Nature.

[3] Cantone I, Marucci L, Iorio F, Ricci MA, Belcastro V (2009). A yeast synthetic network for *in vivo* assessment of reverse-engineering and modeling approaches.. Cell.

[4] Menolascina F, di Bernardo M, di Bernardo D (2010). Design and implementation of a feedback control strategy for IRMA, a novel synthetic gene regulatory network..

[5] Menolascina F, di Bernardo M, di Bernardo D Analysis, design and implementation of a novel scheme for in-vivo control of synthetic gene regulatory networks..

[6] Horak J, Wolf D (1997). Catabolite inactivation of the galactose transporter in the yeast Saccharomyces cerevisiae: ubiquitination, endocytosis, and degradation in the vacuole.. J Bacteriol.

[7] Yano K, Fukasawa T (1997). Galactose-dependent reversible interaction of Gal3p with Gal80p in the induction pathway of Gal4p-activated genes of Saccharomyces cerevisiae.. Proc Natl Acad Sci U S A.

[8] Diep CQ, Tao X, Pilauri V, Losiewicz M, Blank TE (2008). Genetic evidence for sites of interaction between the Gal3 and Gal80 proteins of the Saccharomyces cerevisiae GAL gene switch.. Genetics.

[9] Johnston M, Flick JS, Pexton T (1994). Multiple mechanisms provide rapid and stringent glucose repression of GAL gene expression in Saccharomyces cerevisiae.. Mol Cell Biol.

[10] de Atauri P, Orrell D, Ramsey S, Bolouri H (2004). Evolution of ‘design’ principles in biochemical networks.. Systems Biology, IEE Proceedings.

[11] Ramsey SA, Smith JJ, Orrell D, Marelli M, Petersen TW (2006). Dual feedback loops in the GAL regulon suppress cellular heterogeneity in yeast.. Nat Genet.

[12] Bennett MR, Pang WL, Ostroff NA, Baumgartner BL, Nayak S (2008). Metabolic gene regulation in a dynamically changing environment..

[13] Bornholdt S (2005). SYSTEMS BIOLOGY: Less is more in modeling large genetic networks.. Science.

[14] de Atauri P, Orrell D, Ramsey S, Bolouri H (2004). Evolution of ‘design’ principles in biochemical networks.. IET Sys Bio.

[15] Chen KC, Calzone L, Csikasz-Nagy A, Cross FR, Novak B (2004). Integrative Analysis of Cell Cycle Control in Budding Yeast.. Mol Biol Cell.

[16] Campbell RE, Tour O, Palmer AE, Steinbach PA, Baird GS (2002). A monomeric red fluorescent protein.. Proceedings of the National Academy of Sciences of the United States of America.

[17] Trumbly RJ (1992). Glucose repression in the yeast *Saccharomyces cerevisiae*.. Molecular Microbiology.

[18] Wikswo JP, Prokop A, Baudenbacher F, Cliffel D, Csukas B (2006). Engineering challenges of bioNEMS: the integration of microfluidics, micro- and nanodevices, models and external control for systems biology.. IEE Proceedings Nanobiotechnology,.

[19] McLean JA, Schultz JA, Woods AS, Cole RB (2010). Ion Mobility-Mass Spectrometry for biological and Structural Mass Spectrometry.. Electrospray and MALDI Mass Spectrometry: Fundamentals, Instrumentation, Practicabilities, and Biological Applications: John Wiley and Sons.

[20] Saltelli A, Tarantola S, Chan KPS (1999). A quantitative model-independent method for global sensitivity analysis of model output.. Technometrics.

[21] Saltelli A, Tarantola S, Campolongo F (2000). Sensitivity analysis as an ingredient of modeling.. Statistical Science.

[22] Sobol IM (2001). Global sensitivity indices for nonlinear mathematical models and their Monte Carlo estimates.. Math Comp Simul.

[23] Draper N, Smith H (1981). Applied regression analysis..

[24] Bentele M, Lavrik I, Ulrich M, Stosser S, Heermann DW (2004). Mathematical modeling reveals threshold mechanism in CD95-induced apoptosis.. J Cell Biol.

[25] Varma A, Morbidelli M, Wu H (1999). Parameteric sensitivity in chemical systems..

